# ATP hydrolysis by UPF1 is required for efficient translation termination at premature stop codons

**DOI:** 10.1038/ncomms14021

**Published:** 2016-12-23

**Authors:** Lucas D. Serdar, DaJuan L. Whiteside, Kristian E. Baker

**Affiliations:** 1 Center for RNA Molecular Biology, Case Western Reserve University School of Medicine, Cleveland, Ohio 44106, USA

## Abstract

Nonsense-mediated mRNA decay (NMD) represents a eukaryotic quality control pathway that recognizes and rapidly degrades transcripts harbouring nonsense mutations to limit accumulation of non-functional and potentially toxic truncated polypeptides. A critical component of the NMD machinery is UPF1, an RNA helicase whose ATPase activity is essential for NMD, but for which the precise function and site of action remain unclear. We provide evidence that ATP hydrolysis by UPF1 is required for efficient translation termination and ribosome release at a premature termination codon. UPF1 ATPase mutants accumulate 3′ RNA decay fragments harbouring a ribosome stalled during premature termination that impedes complete degradation of the mRNA. The ability of UPF1 to impinge on premature termination, moreover, requires ATP-binding, RNA-binding and NMD cofactors UPF2 and UPF3. Our results reveal that ATP hydrolysis by UPF1 modulates a functional interaction between the NMD machinery and terminating ribosomes necessary for targeting substrates to accelerated degradation.

## Abstract (web summary)

Nonsense-mediated mRNA decay (NMD) is a quality control pathway that recognizes and degrades transcripts harbouring nonsense mutations. Here the authors show that the ATPase activity of UPF1 mediates functional interactions between the NMD machinery and ribosomes required for efficient ribosome release at premature termination codons.

Quality control checkpoints exist during gene expression to detect and eliminate intermediates lacking integrity or functionality. For messenger RNAs (mRNAs) harbouring a nonsense mutation, premature translation termination precludes the synthesis of a full-length polypeptide and relegates the transcript to rapid degradation via the nonsense-mediated mRNA decay (NMD) pathway^1^,^2^,^3^. Three proteins, UPF1, UPF2 and UPF3, comprise the core components of the NMD machinery and are highly conserved from yeast to humans. UPF1, an RNA-dependent ATPase and member of the SF1 family of RNA helicases, is the only core NMD factor to exhibit catalytic activity and serves as the central driver for targeting nonsense codon-containing mRNA to NMD. Critically, mutation of conserved aspartate and glutamate residues within motif II of the UPF1 helicase domain prevents ATP hydrolysis and renders the NMD pathway inactive^4^. While the ATP-binding and hydrolysis cycle of human UPF1 has been implicated in limiting the association of UPF1 with non-target mRNAs^5^,^6^ and promoting disassembly of proteins from substrates after targeting to NMD^7^,^8^, the precise site of action and function of ATP hydrolysis by UPF1 remain unknown.

The cascade of events during NMD that culminates in the accelerated degradation of a nonsense-containing mRNA begins with a prematurely terminating ribosome. The key question of how the NMD machinery monitors translation and distinguishes between normal and premature termination has been the focus of intense scrutiny. While it is now generally assumed that the NMD machinery communicates with a terminating ribosome through characterized interactions between UPF1 and eukaryotic release factors, eRF1 and eRF3 (refs 1, 9, 10, 11), it has been debated how this interaction is initially established and what events must subsequently transpire to promote accelerated decay of the mRNA. Indeed, while it has been proposed that premature translation termination is inherently aberrant and sufficient to recruit UPF1 to the translation machinery, recent global RNA-binding assays reveal that UPF1 interacts with both normal and nonsense-containing mRNAs and that it binds transcripts in a translation-independent manner^12^,^13^,^14^,^15^. Moreover, although evidence in support of a role for UPF1 in influencing the efficiency of translation termination at nonsense codons has been presented^4^,^16^, the mechanism(s) by which this occurs remain to be established. Finally, despite the fact that cofactors UPF2 and UPF3 are critical in targeting most nonsense-containing mRNA to NMD, their precise functions in the pathway are still ill defined. Thus, our mechanistic understanding of the requirements and consequences of UPF1 interaction with the ribosome on targeting an mRNA to NMD is far from complete.

Herein, we provide evidence that ATP hydrolysis by UPF1 is required for efficient translation termination and ribosome release at premature termination codons. ATPase-deficient mutants of UPF1 in yeast accumulate 3′ RNA decay intermediates bound by ribosomes stalled during premature translation termination that, in turn, impose an impediment to the complete 5′→3′ exonucleolytic degradation of the mRNA. We show that ATP binding and RNA binding by UPF1 and the NMD cofactors UPF2 and UPF3 are required for the function of UPF1 on termination, and provide evidence that in this context, UPF2 functions independently of its ability to induce structural rearrangements within UPF1 that activate its ATP hydrolysis and helicase activities. Our results demonstrate a functional interaction between UPF1 and terminating ribosomes *in vivo*, and reveal a direct role for ATP hydrolysis in modulating the efficiency of translation termination on NMD substrates. These data add to the growing body of evidence that the stability of both normal and aberrant mRNAs is tightly controlled by events directly impacting the efficiency of mRNA translation.

## Results

### 3′ RNA decay intermediates accumulate in UPF1 ATPase mutants

**Figure 1:**
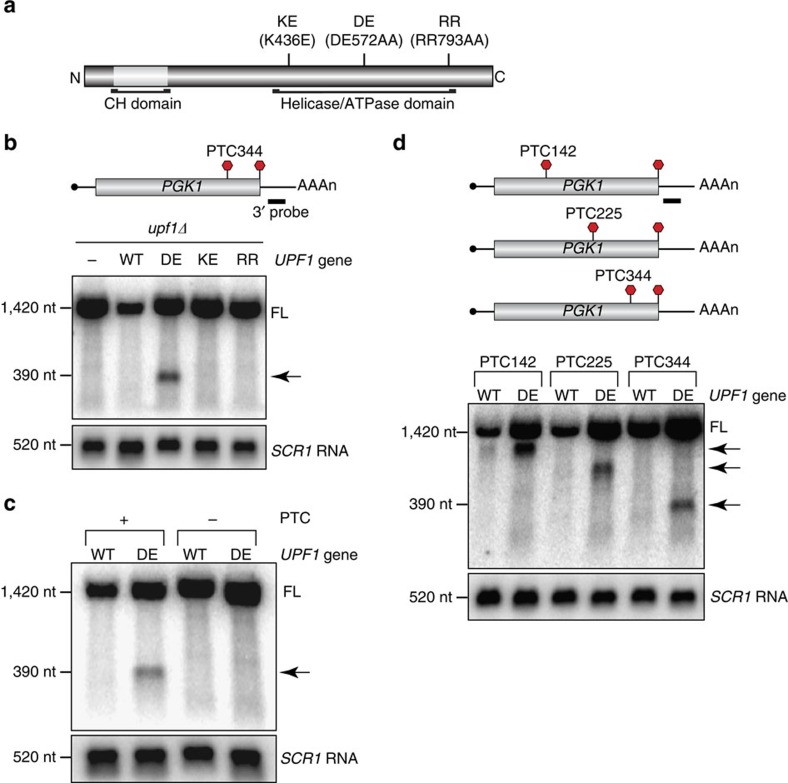
Accumulation of a 3′ RNA fragment from nonsense-containing mRNA in UPF1 ATP hydrolysis mutants. (**a**) Schematic diagram of point mutations within the C-terminal helicase domain of UPF1 that impair ATP binding (K436E), ATP hydrolysis (DE572AA) or RNA binding (RR793AA). The cysteine/histidine-rich domain (CH) within the N-terminus of UPF1 is indicated. (**b**–**d**) Northern blot analysis of *PGK1* reporter mRNA in *upf1*Δ cells (−) complemented with wild-type (WT) or mutant *UPF1* using a probe complementary to the mRNA 3′ end. Reporter mRNA either harboured a PTC within its 416 codon open reading frame (**b**–**d**) or lacked a PTC (**c**; −). Full-length reporter mRNA (FL) and 3′ RNA fragments (arrow) are indicated. RNA levels were normalized to NMD-insensitive *SCR1* RNA. Results are representative of three independent experiments. Nt, nucleotide.

UPF1 mutants with defects in ATP hydrolysis (DE572AA), ATP binding (K436E) or RNA binding (RR793AA; Fig. 1a)^4^ were examined to evaluate the role of these biochemical activities in targeting nonsense-containing mRNA to the NMD pathway in yeast. The steady-state abundance of *PGK1* reporter mRNA harbouring a nonsense or premature termination codon (PTC; at position 344; Fig. 1b, top) was monitored in cells deleted for endogenous *UPF1* (*upf1*Δ) and expressing comparable levels of plasmid encoded wild-type or mutant *UPF1* (Supplementary Fig. 1a). Consistent with targeting of the nonsense-containing reporter to NMD, *PGK1* mRNA abundance was elevated ∼4-fold in cells lacking UPF1 (Fig. 1b; compare full-length RNA in lanes 1 and 2). Moreover, in the presence of all three UPF1 mutants, reporter mRNA abundance was similar to *upf1*Δ cells, confirming observations that these mutations inactivate UPF1 and disable the NMD pathway^4^.

In cells expressing ATPase-deficient UPF1, a rapidly migrating RNA species was detected that was not present in the ATP- or RNA-binding mutants, or in the complete absence of UPF1 (Fig. 1b, arrow). This RNA species corresponds to the 3′ end of *PGK1* mRNA since the probe hybridizes to a unique region within the reporter 3′ untranslated region (UTR), and a probe complementary to the mRNA 5′ end upstream of the PTC failed to detect this species (Supplementary Fig. 1b). Appearance of the smaller RNA species was also dependent on premature translation termination, as *PGK1* reporter mRNA lacking the nonsense codon failed to generate a similar band (Fig. 1c).

Our data reveal distinct functional consequences for inactivation of ATP-binding versus ATP hydrolysis activities of UPF1 on the accumulation of the 3′ RNA fragment (compare DE572AA and K436E mutants; Fig. 1b). This observation is unexpected given that corresponding mutations in human UPF1 (that is, DE636AA and K498A) behave similarly in reports implicating the protein in disassembly of protein complexes from NMD substrates^7^ and in NMD target discrimination^5^. We addressed this anomaly by analysing additional missense mutations at lysine 436 reported to disrupt ATP binding by UPF1 (ref. 4). In all cases, substitution of K436 inactivated UPF1 and eliminated targeting of substrates to NMD (Supplementary Fig. 1c). Consistent with our results when glutamic acid was introduced at this position (that is, K436E), replacement of this residue with either proline or glutamine failed to yield a faster migrating 3′ RNA species. In contrast, insertion of alanine (that is, K436A) led to the appearance of a 3′ RNA fragment similar in size to that formed in cells harbouring ATPase-deficient UPF1 (Supplementary Fig. 1d). Although a direct comparison of the ATP-binding and hydrolysis activities for these mutants is lacking, one simple interpretation of these data is that the UPF1 K436A mutant, while devoid of ATP hydrolysis activity, retains residual *in vivo* ATP-binding capacity (Supplementary Discussion). Consistent with a diminished yet functional ability to bind ATP, the abundance of the 3′ RNA fragment in the K436A mutant was 2.5-fold less than that for the DE572AA mutant, of which the latter exhibits virtually identical *in vitro* ATP binding kinetics as wild-type UPF1 (ref. 17).

To characterize the nature of the RNA product accumulating in UPF1 ATP hydrolysis mutants, reporter mRNAs harbouring a nonsense mutation at a variety of codon positions were analysed (Fig. 1d, top). Notably, 3′ RNA fragments of variable size were detected for mRNA reporters in the presence of ATPase-deficient UPF1, with sizes coincident with the length of RNA extending from the nonsense codon to the mRNA 3′ end (Fig. 1d, bottom). The accumulation of 3′ RNA products was independent of the mRNA analysed, as nonsense-containing *GFP* mRNA also gave rise to a similar PTC position-dependent pattern of 3′ RNA fragments (Supplementary Fig. 2). The appearance of 3′ RNA fragments, whose size is dictated by nonsense codon position, suggests that in the absence of ATP hydrolysis by UPF1, a block to decay of nonsense-containing mRNA occurs at or near the site of premature translation termination.

**Figure 2:**
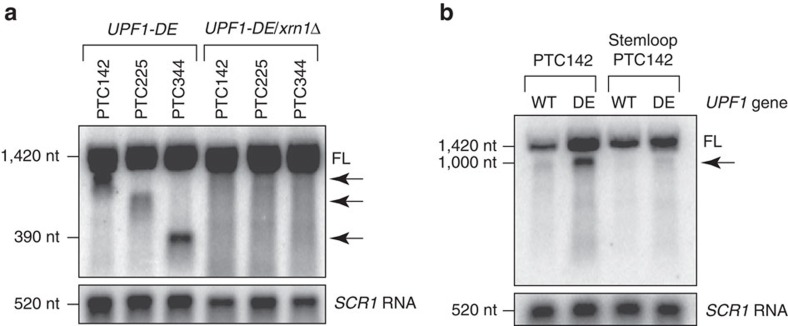
3′ RNA fragments are decay intermediates dependent on XRN1 activity and translation. (**a**,**b**) Northern blot analysis of PTC-containing *PGK1* reporter mRNA in *upf1*Δ cells expressing ATPase-deficient UPF1 in the presence (*UPF1-DE*) or absence of XRN1 (*UPF1-DE/xrn1*Δ). (**b**) Reporter mRNA inhibited for translation by a 5′ stemloop in *upf1*Δ cells expressing either wild-type (WT) or ATPase-deficient (DE) UPF1. Full-length reporter mRNA (FL) and 3′ RNA fragments (arrow) indicated. RNA levels were normalized to NMD-insensitive *SCR1* RNA. Results are representative of three independent experiments. Nt, nucleotide.

Degradation of mRNA by either the canonical decay pathway or by NMD in yeast proceeds via decapping of the RNA 5′ end followed by 5′→3′ digestion catalysed by the cytoplasmic exoribonuclease XRN1 (refs 18, 19). Processive activity of XRN1 *in vivo* is inhibited by strong RNA secondary structure^20^ or by translocating ribosomes during the process of co-translational mRNA decay^21^,^22^. Critically, 3′ RNA species that accrue in UPF1 ATPase mutants were lost in cells lacking XRN1 (*xrn1*Δ;Fig. 2a), indicating that the fragments represent *bona fide* products of 5′→3′ exonucleolytic activity. Moreover, mRNA translation is required for accumulation of the 3′ RNA decay product (Fig. 2b) as demonstrated by loss of the fragment from *PGK1* reporter mRNA harbouring a stable stemloop structure in its 5′ UTR that represses translation >100-fold^23^ (Supplementary Fig. 3) but does not impede XRN1 activity^18^.

### 3′ RNA decay intermediates are ribosome bound

**Figure 3:**
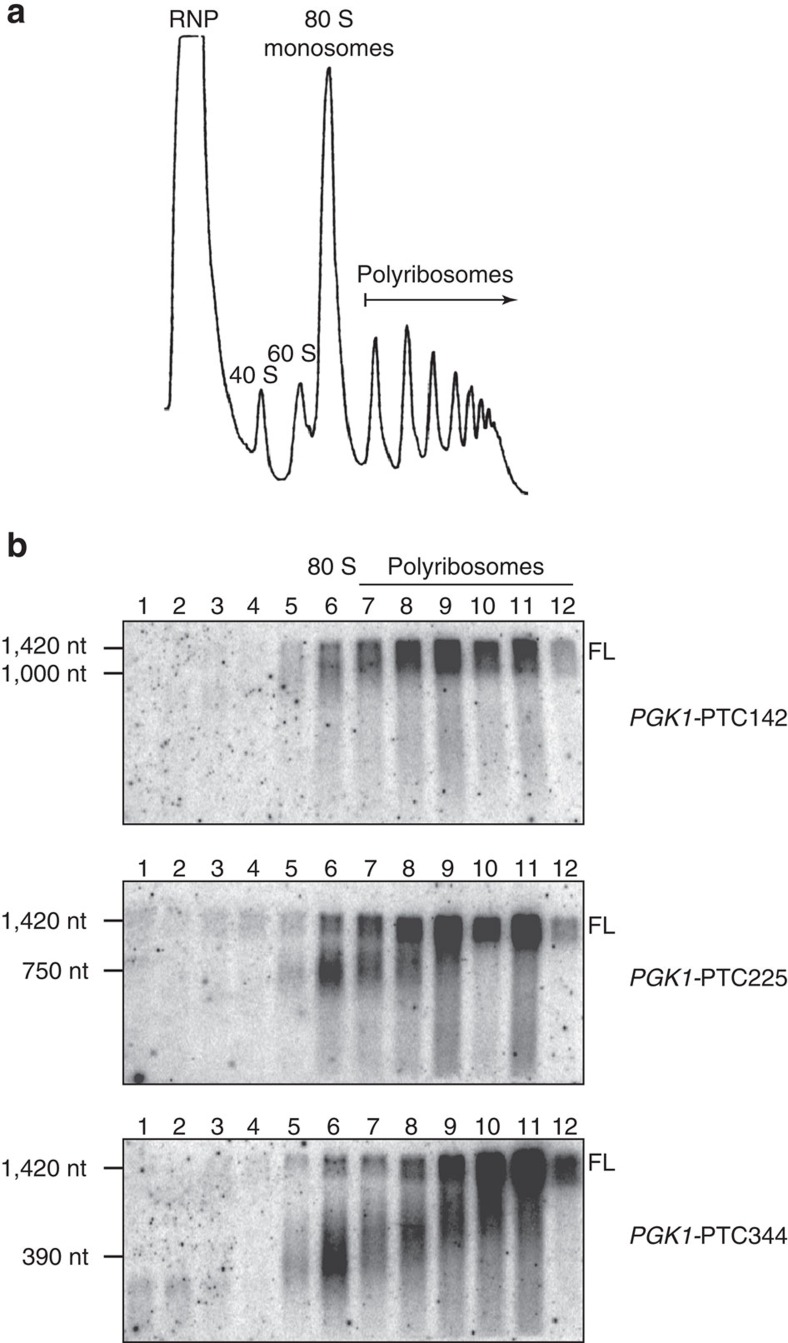
3′ RNA decay fragments are ribosome bound. (**a**) Ultraviolet absorbance trace from polyribosome analysis of cells expressing ATPase-deficient UPF1 and one of three PTC-containing *PGK1* reporter mRNAs; ribosome-free RNA (RNP), 40S, 60S and 80S ribosomal subunits, and polyribosomes are indicated. (**b**) Northern blot analysis of PTC-containing *PGK1* reporter mRNA from each gradient fraction. Full-length reporter mRNA (FL) is indicated. Results are representative of three independent experiments. Nt, nucleotide.

Our data suggest that the 3′ RNA fragments that arise from nonsense-containing mRNA in ATPase-deficient UPF1 mutants accumulate due to a block in 5′→3′ degradation caused by a ribosome stalled during premature translation termination. To evaluate directly whether the 3′ RNA decay products are ribosome bound, lysates from cells expressing one of three nonsense codon-containing *PGK1* reporter mRNAs were subjected to sucrose density gradient centrifugation (that is, polyribosome analysis). Using this method, ribosome-free RNA (that is, RNP) was separated from transcripts associated with one, or multiple, ribosomes^24^ (Fig. 3a). Analysis of RNA isolated from gradient fractions showed that for each of the nonsense-containing *PGK1* reporter mRNAs, a 3′ RNA species cosedimented precisely with 80S monosomes (Fig. 3b, lane 6), and its size corresponded to the 3′ RNA decay fragment observed at steady state. Moreover, 3′ RNA fragments of increasing size were detected in progressively denser polyribosome fractions, indicative of a build-up of a mounting number of ribosomes along the transcript and co-translational decay of the nonsense-containing reporter mRNA^21^ (Fig. 3b, lanes 7–12 and Supplementary Fig. 4a). Notably, 3′ RNA fragments were not readily detected in RNP fractions (Fig. 3b, lanes 1–3), as would be expected if they were ribosome-free, RNA–protein complexes that failed to be disassembled.

Our data demonstrate that 3′ RNA products of nonsense-containing mRNAs are associated with a single ribosome bound at or near the site of premature termination, and indicate that in the presence of ATPase-deficient UPF1, translation is impeded such that ribosomes fail to terminate properly and display prolonged association with the mRNA. Consistent with a conclusion that the 3′ RNA fragments accumulate as a consequence of a defect in translation termination and a stalled ribosome presenting a block to complete transcript degradation, depletion of RLI1, an ATP-binding cassette-type ATPase required for stimulating peptide release and ribosome subunit dissociation during translation termination^25^,^26^, gave rise to 3′ RNA fragments comparable to those observed in cells expressing ATPase-deficient UPF1 (Supplementary Fig. 4b–d; also see Supplementary Discussion).

### Requirements for UPF1 activity on terminating ribosomes

**Figure 4:**
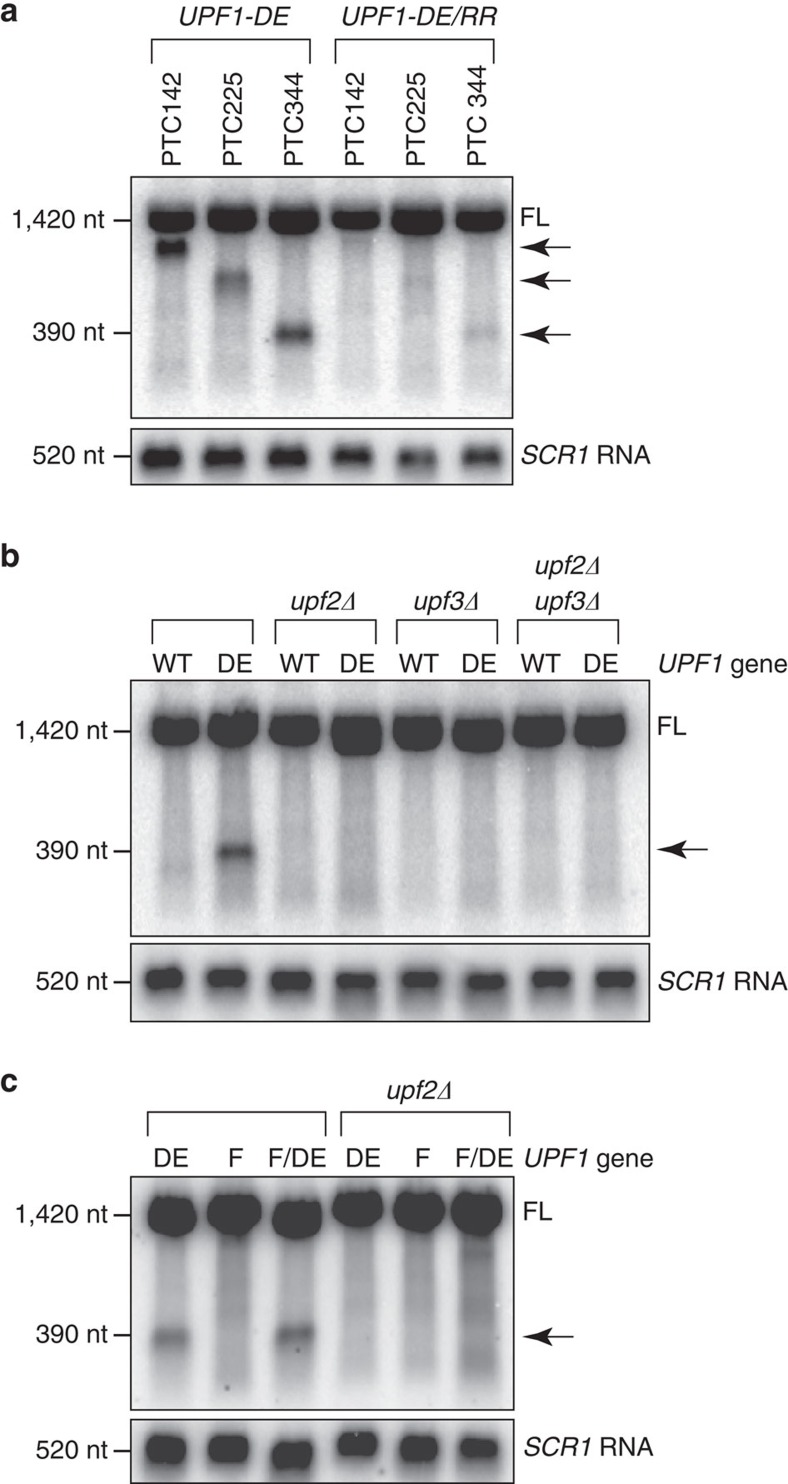
UPF1 function in premature translation termination requires RNA binding and cofactors UPF2 and UPF3. (**a**–**c**) Northern blot analysis of PTC-containing *PGK1* reporter mRNA. (**a**) *upf1*Δ cells complemented with either ATPase-deficient UPF1 (*UPF1-DE*) or the same mutant also lacking RNA-binding activity (*UPF1-DE/RR*). (**b**) *PGK1-PTC344* reporter mRNA in *upf1*Δ cells expressing wild-type (WT) or mutant UPF1 (DE) and also lacking UPF2 (*upf2*Δ), UPF3 (*upf3*Δ) or both (*upf2*Δ*upf3*Δ). (**c**) *PGK1-PTC344* reporter mRNA in *upf1*Δ cells expressing ATPase-deficient UPF1 (DE), mutant UPF1 relieved for allosteric inhibition of ATPase and helicase activities (F), or UPF1 harbouring both mutations (F/DE) in cells either with or without *UPF2* (*upf2*Δ). Full-length reporter mRNA (FL) and 3′ RNA fragments (arrow) are indicated. RNA levels were normalized to NMD-insensitive *SCR1* RNA. Results are representative of three independent experiments. Nt, nucleotide.

Biochemical and structural studies on yeast and human UPF1 implicate both RNA binding and an interaction with NMD cofactors UPF2 and UPF3 in stimulating its ATPase and helicase activities^27^,^28^,^29^. In light of the fact that UPF1 DE572AA is unable to catalyse ATP hydrolysis, we evaluated whether these activators are therefore required for the accumulation of 3′ RNA decay fragments from nonsense-containing reporter mRNAs in the mutant. Introduction of secondary mutations into ATPase-deficient UPF1 that render the protein deficient in RNA binding (that is, RR793AA)^4^ almost completely abolished accumulation of the 3′ decay fragment (Fig. 4a), indicating that an interaction between UPF1 and its RNA substrate is a prerequisite for its ability to inhibit translation termination.

We also found that 3′ RNA decay fragments failed to accumulate in UPF1 ATPase mutants lacking UPF2, UPF3 or both (Fig. 4b), validating the function of ATP hydrolysis by UPF1 on translation termination as a *bona fide* activity of the NMD machinery. Moreover, the role for UPF2 depended on its ability to interact with the N-terminal cysteine/histidine-rich domain of UPF1, as deletion of the domain (that is, ΔCH) or introduction of a point mutation in this region that disrupts the interaction (that is, C62Y)^30^ abolished accumulation of the 3′ RNA decay fragment in UPF1 ATPase mutants (Supplementary Fig. 5a,b). Importantly, the function of UPF2 was distinct from its ability to induce structural rearrangements within UPF1 that stimulate ATPase activity *in vitro*, since a mutation that alleviates allosteric regulation by UPF2 (that is, UPF1 F131E)^29^ failed to restore accumulation of the 3′ RNA decay fragment in cells lacking UPF2 (Fig. 4c; also see Supplementary Discussion). Together, these data reveal a requirement for RNA binding and NMD cofactors UPF2 and UPF3 in modulating the function of UPF1 on premature translation termination, and suggests that, *in vivo*, UPF2 plays an additional role in regulating UPF1 that extends beyond inducing conformational changes necessary for stimulating UPF1 helicase activity^8^,^28^,^29^.

## Discussion

We present a number of observations that collectively serve to demonstrate that RNA fragments seen in cells expressing ATPase-deficient UPF1 are 3′ decay products of nonsense-containing mRNA harbouring a single ribosome bound at or near the PTC that accumulate as a result of a block to 5′→3′ exonucleolytic digestion by XRN1. These 3′ RNA decay fragments provide clear evidence that UPF1 directly influences translation termination on nonsense-containing mRNAs *in vivo* and reveal a site of action and novel function for ATP hydrolysis by UPF1 in NMD.

**Figure 5:**
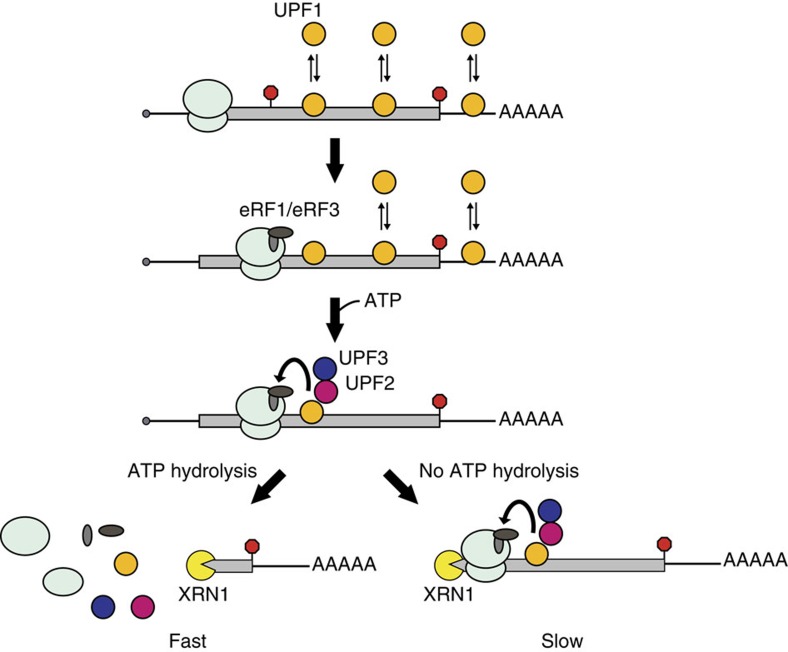
ATP hydrolysis by UPF1 promotes efficient translation termination on nonsense-containing mRNAs. Premature translation termination allows UPF1 to remain associated with transcripts at sites downstream of the PTC and in a 3′ UTR length-dependent manner. ATP binding by UPF1, in conjunction with cofactors UPF2 and UPF3, promotes association of the NMD machinery with a terminating ribosome, mediated through interaction with translation release factors eRF1 and eRF3. For wild-type UPF1, ATP hydrolysis promotes rapid disassembly of the trimeric complex (ribosome, RNA and UPF proteins) leading to efficient translation termination and release of the ribosome and NMD factors from the RNA. How this event is communicated to the decay machinery to accelerate decapping and turnover of the mRNA remains unclear (bottom left; Fast). Failure to catalyze ATP hydrolysis leads to a defect in translation termination and a kinetic stall in ribosome release, presenting a block to XRN1-mediated 5′→3′ exonucleolytic decay and, critically, failure to trigger rapid turnover of the mRNA (bottom right; Slow).

Our discovery that ATP hydrolysis by UPF1 plays a critical role in translation termination and our description of events required for this activity provide mechanistic insight into NMD substrate discrimination and degradation (Fig. 5). We found that RNA binding is necessary for mutant UPF1 to cause 3′ RNA fragment accumulation (Fig. 4a), and suggest that the binding of UPF1 to mRNA represents an early and, perhaps, initial step in NMD that, in turn, serves to facilitate interaction between the NMD machinery and the terminating ribosome. A view in which the initial association between UPF1 and its substrate is mediated through direct RNA binding is, however, contrary to models that posit that UPF1 is recruited to transcripts by elongating or terminating ribosomes (for a review, see ref. 1). Notwithstanding, recent global RNA-binding studies support the premise that UPF1 associates with transcripts in a translation-independent manner^14^,^15^. Moreover, a paucity of UPF1-binding sites within mRNA coding regions and at stop codons in cells undergoing active translation argues that translocating ribosomes function not in recruiting but, rather, displacing UPF1 from RNA^14^,^15^. Thus, a favoured scenario emerges in which UPF1 associates stochastically with all cytoplasmic RNA transcripts, but that for nonsense-containing mRNA, premature translation termination prevents elongating ribosomes from removing UPF1 from downstream RNA regions that would otherwise be involved in protein coding. In comparison with mRNA lacking a PTC, UPF1 association with nonsense-containing transcripts would, therefore, be expected to be enhanced and proportional to the length of RNA downstream of the PTC. Indeed, UPF1 binds preferentially to PTC-containing mRNAs and does so in a 3′ UTR length-dependent manner^12^,^13^,^31^. Moreover, this graded association between UPF1 and RNA appears to have a functional consequence to the fate of the transcript, as mRNAs with 5′-proximal PTCs are targeted to NMD more efficiently than those with 3′-proximal PTCs^32^,^33^,^34^,^35^.

Communication between RNA-bound UPF1 and the terminating ribosome is predicted to occur through characterized and conserved interactions with the translation release factors, eRF1 and eRF3 (refs 9, 10, 11). We observe loss of the 3′ RNA decay fragments in UPF1 ATPase mutants in the absence of UPF2 and/or UPF3 (Fig. 4b), leading us to propose that failure of mutant UPF1 to inhibit termination in these cells reflects a role for the cofactors in stabilizing a complex that comprises the RNA, NMD machinery and terminating ribosome. While, in principle, UPF2 and UPF3 could act to promote the association of UPF1 with the RNA, as suggested by their role as components of mammalian exon junction complexes^28^,^36^, we favour a view that these NMD factors serve to enhance the interaction of UPF1 with the terminating ribosome. In support of this, UPF2 and UPF3 each physically interact with eRF3 (at sites distinct from that bound by UPF1)^37^,^38^, and the affinity of UPF1 for RNA *in vitro* is, in fact, diminished in the presence of UPF2^28^. Notably, we found that a point mutation in UPF1 sufficient to alleviate the requirement for UPF2 in stimulating UPF1 helicase activity *in vitro* failed to restore the ability of 3′ RNA decay fragments to accumulate in cells lacking UPF2 (Fig. 4c). This latter observation was unexpected and predicts a novel role for UPF2 in NMD independent of inducing conformational changes in UPF1 necessary for its activation. It remains to be experimentally established whether this purported function for UPF2 involves promoting interactions between the NMD machinery and the terminating ribosome.

Our finding that UPF1 mutants deficient in ATP hydrolysis but not ATP binding accumulate 3′ RNA decay intermediates (Fig. 1b and Supplementary Fig. 1d) suggests that only ATP-bound UPF1 interacts efficiently with the terminating ribosome. Consistent with this, association of RNA-bound UPF1 with eRF3 *in vitro* requires ATP^10^ and is reduced for a UPF1 mutant predicted to lack ATP-binding activity (K498Q in human UPF1)^9^. We speculate that while ATP binding by UPF1 is necessary to facilitate the interaction between RNA-bound UPF1 and the terminating ribosome, ATP hydrolysis is critical for the timely resolution of this complex. Indeed, our data strongly support the interpretation that failure of UPF1 to hydrolyse ATP delays ribosome dissociation from the RNA and blocks 5′→3′ decay of a nonsense codon-containing transcript (Fig. 5, bottom right). By controlling the dissociation kinetics of this complex, ATP hydrolysis would, moreover, govern the association of UPF1 with an NMD substrate, albeit indirectly. This premise provides an alternative explanation for why ATPase mutants of human UPF1 co-immunoprecipitate increased amounts of PTC-containing mRNA and a 3′ RNA decay fragment generated from NMD-mediated endonucleolytic cleavage^5^,^7^.

It has been proposed that 3′ decay intermediates observed for UPF1 mutants deficient in either ATP binding or ATP hydrolysis in human cells accumulate due to a block in decay caused by proteins (including UPF1 itself) that failed to be remodelled from the RNA downstream of the PTC^7^. While ATPase mutants lack 5′→3′ helicase activity^4^, we offer three lines of evidence in support of the interpretation that it is a stalled ribosome and not general RNA-binding proteins retained on the RNA that blocks XRN1-mediated exonucleolytic decay of the RNA. First, 3′ RNA fragments found in ATPase-deficient UPF1 in yeast cosediment with 80S monosomes, consistent with these decay intermediates being bound by a single ribosome. Second, 3′ RNA decay fragments are coincident with the length of the mRNA from the PTC to 3′ end and are generally homogenous in size, indicating that the block to 5′→3′ decay occurs at a fixed site at or near the premature termination event. Interestingly, global RNA-binding analysis of wild-type or ATPase-deficient UPF1 in mammalian cells by crosslinking and immunoprecipitation show that UPF1 is positioned throughout mRNA 3′ UTRs^14^,^15^, predicting that 3′ decay fragments arising from UPF1 blockage would be heterogeneous in size. Third, while we and others have established that 5′→3′ degradation by XRN1 can be efficiently impeded by a ribosome^21^,^22^, we are unaware of evidence demonstrating that UPF1, or any other RNA-bound protein (independent of an association with a stable RNA structure), is sufficient to inhibit XRN1 activity either *in vivo* or *in vitro*.

We note that the 3′ RNA decay fragments observed in ATPase mutants were absent in cells harbouring wild-type UPF1 (Fig. 1b), and attribute this to differences in the activity of wild-type and ATPase-deficient UPF1 on a terminating ribosome. We propose that both wild-type and mutant UPF1 proteins impinge on premature termination events, but that only in the presence of catalytically inactive UPF1 does the complex persist to an extent sufficient to block XRN1 activity and accumulate 3′ decay fragments detectable by northern blot. Consistent with wild-type UPF1 also engaging with the translation machinery during termination, impaired ribosome release at premature termination codons dependent on UPF1 has been detected in yeast extracts using toeprint assays^16^.

Our data place ATP hydrolysis by UPF1 at a pivotal point in the NMD pathway—after a functional complex between UPF1 (in conjunction with UPF2 and UPF3), the mRNA and a terminating ribosome has been established, and centred around events at the ribosomal A site that ultimately communicate information which leads to accelerated turnover of the mRNA. It remains to be determined whether the energy released upon ATP hydrolysis by UPF1 promotes efficient termination by stimulating peptide release or the subsequent recycling of ribosomes from the mRNA. Notwithstanding, the inability of ATPase-deficient UPF1 to target nonsense-containing mRNA to accelerated decay indicates that, regardless of its precise role, UPF1 function in translation termination is monitored and critical to mediate accelerated decay of the transcript. Thus, an important goal for the field will be to elucidate how modulation of termination by UPF1 is communicated to the degradation machinery, which in yeast leads to accelerated decapping of the mRNA 5′ end. Despite the lack of a complete molecular understanding, the function of UPF1 on the terminating ribosome adds to the growing list of events monitored at the ribosomal A site, including the rate of cognate tRNA occupancy^39^ and binding of molecular mimics (that is, DOM34 and SKI7)^40^,^41^, which lead to profound effects on mRNA stability for both normal and aberrant RNA transcripts. Thus, it is becoming increasingly clear that the ribosome acts not only as a molecular decoder but also as an exquisite sensor coupling perturbations in the efficiency of translation of an mRNA to its stability.

## Methods

### Yeast culture and standard methods

Yeast strains used in this study are listed in Supplementary Table 1. Yeast cultures were grown at 30 °C with shaking at 250 r.p.m. in synthetic medium supplemented with appropriate amino acids and either 2% glucose (SD) or 2% galactose and 1% sucrose (SGS). RNA isolation, polyribosome analysis and northern blotting were performed as described previously^24^.

### Plasmid construction

Plasmids and oligodeoxynucleotides used in this study are listed in Supplementary Tables 2 and 3, respectively. Plasmid-encoded PTC-containing *PGK1* reporters were generated by site-directed mutagenesis of pJC408 using oKB891/oKB892 to generate pKB581 (PTC142), oKB397/oKB398 to generate pKB589 (PTC344) or oKB908/oKB909 to generate pKB593 (PTC225). *SL-PGK1-PTC142* (pKB590) was generated by site-directed mutagenesis of pJC424 using oKB891/oKB892. PTC-containing *GFP* reporters were generated by site-directed mutagenesis of pKB290 using oKB227/oKB228 to generate pKB311 (PTC135) or oKB229/oKB230 to generate pKB312 (PTC200). Constitutively expressed *GFP-PTC135* (pKB510) was generated by excision of the *GAL1* promoter from pKB311 with *Spe*I and *Bam*HI and replacing it with the *TDH3* promoter from pKB209. To construct wild-type *UPF1-HA* plasmid (pKB556), DNA from yKB494 was PCR amplified using oKB736/oKB737 and blunt-end ligated into the *Sma*I site of pKB105. To construct wild-type *UPF1* plasmid (lacking HA tag, pKB598), DNA was PCR amplified from yKB154 using oKB849/oKB850, and cloned into *Pst*I and *Bam*HI sites in pKB102. Mutant alleles of *UPF1-HA* or *UPF1* were generated by site-directed mutagenesis of either pKB556 or pKB598, using oKB823/oKB824 (DE572AA; pKB576 and pKB607), oKB825/oKB826 (K436E; pKB578), oKB827/oKB828 (RR793AA; pKB579), oKB829/oKB830 (C62Y; pKB642), oKB1144/oKB1145 (F131E; pKB640), oKB1324/oKB1325 (K436A; pKB692), oKB1326/oKB1327 (K436P; pKB690) or oKB1328/oKB1329 (K436Q; pKB691). *UPF1* double mutants (pKB610, pKB621, pKB641) were generated from pKB607 using the above-indicated oligonucleotides for site-directed mutagenesis for each specific mutation. To construct *UPF1-*Δ*CH* (pKB638), *Spe*I sites bracketing the cysteine/histidine-rich (CH) domain were introduced by site-directed mutagenesis using oKB1031/oKB1032 (5′ site; codon 62) or oKB1097/oKB1098 (3′ site; codon 212) using pKB598 as a template. The CH domain was then excised by digestion with *Spe*I, and the resulting linearized plasmid religated to generate pKB638. To construct *UPF1-*Δ*CH-DE572AA* (pKB645), pKB638 was used as a template for site-directed mutagenesis using oKB823/oKB824.

### Protein isolation and western blot analysis

Yeast cultures expressing chromosomally encoded, *UPF1-HA*, *3HA-RLI1*, or plasmid-encoded, *UPF1-HA*, alleles were grown to mid-log phase and flash frozen on dry ice. Cell pellets were heated in 5 M urea for 2 min at 95 °C, and lysed by mechanical disruption with glass beads by vortexing for 5 min. Solution A was added to lysates (125 mM Tris-HCl, pH 6.8, 2% SDS) and samples vortexed for 1 min, followed by heating to 95 °C for 2 min. Glass beads and cellular debris were cleared from lysates by centrifugation at 13,200 r.p.m. for 4 min. Equivalent optical density units (*A*260) of lysate in 1 × SDS sample buffer (125 mM Tris-HCl, pH 6.8, 2% SDS, 100 mM DTT (dithiothreitol), 10% glycerol, 0.05% bromphenol blue) were separated on 7.5% Bis-Tris polyacrylamide gels by electrophoresis in 1 × SDS running buffer (25 mM Tris base, 192 mM glycine, 0.1% SDS). Proteins were transferred to PVDF (polyvinylidene difluoride) transfer membrane (Thermo Scientific; 88,518) in 1 × transfer buffer (25 mM Tris base, 192 mM glycine, 20% methanol) by electroblotting at 4 °C for 2 h at 250 mA. Membranes were blocked (5% milk powder in 1 × TBS (Tris-buffered saline)/0.1% Tween-20) overnight at 4 °C and proteins were detected by incubating with primary antibodies (mouse monoclonal α-HA 1:5,000 (Covance; MMS-101P), or mouse monoclonal α-PAB1 1:10,000 (Encore Biotechnology; MCA-1G1) and secondary antibodies (goat α-mouse immunoglobulin G horseradish peroxidase 1:5,000 (Santa Cruz; sc-2005) in blocking buffer for 1 h at room temperature. Between incubations, membranes were washed with 1 × TBS/0.1% Tween-20 three times each for 15 min. Signals were detected by chemiluminescence using Blue Ultra Autorad film (GeneMate; F-9029).

### Conditional depletion of *RLI1*

Chromosomally encoded *RLI1* was placed under control of the *GAL1* promoter using standard methods^42^. Briefly, the His3MX6-P_GAL1_-3HA insertion cassette was PCR amplified using Phusion High Fidelity DNA polymerase (NEB; M0530S) and oKB1123/oKB1124. PCR products were run on 1% agarose gels, purified using Zymoclean Gel DNA Recover Kits (Zymo Research; D4002), and transformed into wild-type yeast (yKB154). For depletion experiments, *P*_*GAL1*_*-3HA-RLI1* cells expressing plasmid-encoded *GFP-PTC135* were inoculated at an initial density of OD_600_=0.02 in SGS media (lacking uracil), and grown at 30 °C for 12 h. Cells were pelleted by centrifugation at 4,000 r.p.m. for 4 min, washed once in synthetic media lacking sugar and resuspended in glucose-containing media (SD lacking uracil) to an OD_600_=0.1. Cells were grown at 30 °C for 10 h and aliquots were removed and collected by centrifugation every 2 h. Cell aliquots were flash frozen on dry ice for downstream analysis of RLI1 protein and reporter mRNA expression by western and northern blotting, respectively.

### Data availability

The data that support the findings of this study are available from the corresponding author on request.

## Additional information

**How to cite this article:** Serdar, L. D. *et al*. ATP hydrolysis by UPF1 is required for efficient translation termination at premature stop codons. *Nat. Commun.* **7,** 14021 doi: 10.1038/ncomms14021 (2016).

**Publisher's note:** Springer Nature remains neutral with regard to jurisdictional claims in published maps and institutional affiliations.

## Supplementary Material

Supplementary Information Supplementary Figures, Supplementary Tables, Supplementary Discussion and Supplementary References
