# Supplementary material for: ATP hydrolysis by UPF1 is required for efficient translation termination at premature stop codons
Source: Nat Commun. 2016 Dec 23;7:14021. doi: 10.1038/ncomms14021 (PMC5196439; doi:10.1038/ncomms14021)

Supplementary Discussion

ATP binding by UPF1 is required for the functional interaction with terminating ribosomes

Our observation that 3' RNA decay fragments detected in an ATP hydrolysis mutant of yeast UPF1 (i.e. DE572AA) do not also accumulate in cells expressing ATP binding-deficient UPF1 (K436E) was unexpected in the context of results that similar mutations in human UPF1 behave alike in studies evaluating their role in mRNP remodeling[1] and NMD target discrimination[2]. In an attempt to understand this discrepancy, three additional missense mutations were introduced at lysine position 436 – alanine, proline, or glutamine - all of which have been previously reported to inactivate the ATP binding activity of yeast UPF1[3]. Consistent with the work of Weng and colleagues in characterizing these mutants, we show that each is defective in targeting PTC-containing mRNA to NMD as demonstrated by the increased steady-state abundance of the endogenous NMD substrate, *CYH2* pre-mRNA[4] (Supplementary Fig. 1c, compare mutants to WT). Notably, consistent with our results with mutant UPF1 encoding glutamic acid at position 436 (i.e. K436E), we did not observe a 3' RNA decay intermediate in mutants where lysine was replaced with either proline or glutamine (K436P and K436Q, respectively; Supplementary Fig. 1d). In contrast, introduction of alanine did result in accumulation of an RNA intermediate similar in size to that formed in cells harboring ATPase-deficient UPF1 (compare DE and KA samples).

Our observation that four inactivating mutations in UPF1 that introduce different substitutions at lysine 436 each predicted to impair ATP binding did not behave similarly in our assay was unexpected. It is important to note, however, that not all of these UPF1 mutants have been characterized biochemically for their ATP binding activities, and that the initial analysis of these mutants was limited to demonstrating that they are defective in targeting substrates to NMD in yeast[3]. Critically, rigorous measurement of ATP binding activity has only been reported for one of the four mutants, K436Q[5], suggesting that the kinetics of ATP binding by the K436A mutant (or the others) has never been experimentally determined. Notably, the K436Q mutation was found to reduce the affinity of UPF1 for ATP 16-fold compared to wildtype, while ATP binding by the UPF1 DE572AA mutant was virtually identical to wildtype[5].

One simple interpretation of our data shown in Supplementary Fig. 1d is that the K436A mutant retains residual ATP binding activity *in vivo* that is sufficient to mediate the function of UPF1 on a prematurely terminating ribosome. We note that human UPF1 harboring the corresponding mutation (replacement of lysine 498 to alanine; K498A) retains ~5% ATP binding activity as measured by a semi-quantitative, *in vitro* steady-state binding assay[6]. We speculate that this residual ATP binding activity exhibited by the hUPF1 K498A mutant *in vitro* is sufficient to provide functional ATP binding activity *in vivo*, albeit at a reduced level. Consistent with this, the abundance of the 3’ RNA decay fragments in cells expressing the UPF1 K436A mutant are 2-3 fold lower then levels found in the DE572AA mutant (Supplementary Fig. 1d).

Our findings are consistent with ATP binding and ATP hydrolysis activities of UPF1 having distinct functions in modulating translation termination at a PTC.

Additionally, our data reveal that while a number of missense mutations in UPF1 predicted to block ATP binding all function to inactivate the NMD pathway, they are not equivalent. Given the observation that the molecular phenotype of the UPF1 K436A mutant is distinct from variants with three other substitutions at this position calls into question the generality of conclusions drawn from experiments examining this particular mutant. It is important to highlight that a number of studies on the role of ATP binding and hydrolysis by human UPF1 in NMD have employed the K498A mutant[1,2]; based on our findings in yeast employing multiple mutations at the conserved residue, it will be of interest to assess additional mutations in hUPF1 to determine if they, too, exert different outcomes on recognition and/or degradation of NMD substrates, and establish whether ATP binding and ATP hydrolysis also have distinct functions in this pathway in mammals.

Depletion of RLI1 inhibits ribosome recycling and leads to accumulation of 3' RNA decay fragments similar to those observed in UPF1 ATPase mutants

Polyribosome analysis demonstrated that 3' RNA decay fragments accumulating in cells expressing ATPase-deficient UPF1 co-sediment with 80S ribosomes, indicating that they are bound by a single ribosome (Fig. 3b). Additionally, progressively larger RNA decay fragments were observed to sediment in increasingly dense fractions of the sucrose gradient, consistent with the piling up of a heightened number of ribosomes upstream of the nonsense codon that impose an ever more 5'-proximal block to XRN1 activity during co-translational decay of the mRNA[7,8] (Supplementary Fig. 4a). To lend support to our conclusion that the observed 3' RNA decay fragments result from a

ribosome stalled during translation termination which, in turn, present a block to 5' → 3' decay, we examined whether a defect in translation termination generated by an independent mechanism would also result in the accumulation of 3' RNA fragments.

As a means to demonstrate that inhibition of translation termination is sufficient to cause the accumulation of 3' RNA decay fragments, termination was blocked by depletion of RLI1, a conserved ABC-type ATPase required for stimulating peptide release *in vitro* and ribosome subunit dissociation during translation termination *in vivo*[9,10]. Similar to published methods[11] chromosomally-encoded *RLI1* was placed under control of a galactose-inducible promoter (P_{GAL1}-*3HA-RLI1*) and expression inhibited by growth of cells in media lacking galactose (see Methods). Eight to ten hours after inhibition of transcription, RLI1 protein abundance was <1% of steady-state levels and culture growth was dramatically impaired, consistent with an essential role for RLI1 in cell viability[11] (Supplementary Fig. 4b,c). Notably, depletion of RLI1 resulted in the accumulation of a 3' RNA fragment from PTC-containing *GFP* reporter mRNA comparable to the fragment generated in cells expressing ATPase-deficient UPF1 (Supplementary Fig. 4d). These data demonstrate that inhibition of translation termination is sufficient to lead to accumulation of 3' RNA decay fragments, strongly supporting our interpretation that the RNA fragments detected in UPF1 mutants arise as a consequence of a stalled ribosome caused by failure of UPF1 to hydrolyze ATP.

We note that the RNA fragments generated upon depletion of RLI1 are heterogeneous and reduced in size relative to the 3' RNA decay fragment in UPF1 mutant cells (Supplementary Fig. 4d). This difference is likely due to transit of ribosomes past termination codons to various positions within mRNA 3' UTRs, as

previously observed by ribosome profiling in cells depleted for RLI1[11]. Such transited ribosomes would permit XRN1 to degrade the transcript beyond the nonsense codon leading to the production of the smaller and heterogeneous 3’ RNA decay fragments, as we observe.

Direct binding of UPF1 by UPF2 is required for UPF1 function in translation termination

We observe a requirement for UPF2 (and UPF3) in the accumulation of 3’ RNA decay fragments in ATPase-deficient UPF1 mutants (Fig. 4B). To assess whether the function of UPF2 is mediated through direct binding to UPF1, two modifications were introduced into UPF1 previously shown to be sufficient to disrupt the physical interaction between these two proteins[12,13]. Notably, either deletion of the entire CH-domain of UPF1 (amino acids 62-212) or a point mutation within this region (C62Y) resulted in the loss of the 3’ RNA decay fragments when introduced into ATPase-deficient UPF1 mutants (Supplementary Fig. 5). These results show that the requirement for UPF2 in UPF1 function is mediated through a direct interaction with UPF1 via its N-terminal CH domain.

Biochemical and structural studies reveal that UPF1 undergoes a conformational rearrangement upon UPF2 binding that alleviates allosteric inhibition caused by an intramolecular interaction between the UPF1 CH and helicase domains and results in enhanced ATPase and helicase activities *in vitro* [14]. The intramolecular interaction within UPF1 is mediated, in part, by a conserved phenylalanine residue (i.e. F131) located at the interface; importantly, mutation of this residue to glutamic acid has been

shown to destabilize contact between the two domains and bypass the requirement for UPF2 binding in activating UPF1[14]. We reasoned, therefore, that introducing the F131E mutation into ATPase-deficient UPF1 may be sufficient to overcome the requirement for UPF2 in the function of mutant UPF1 on termination. Notably, in cells expressing UPF2, introduction of the F131E mutation did not alter the ability of ATPase-deficient UPF1 to promote 3' RNA fragment accumulation in the presence of UPF2 (Fig 4c, lane 3). However, the mutation also did not bypass of the requirement for UPF2 in our assay (Fig. 4c, lane 6). While it is possible that the F131E mutation does not impose a consequence on UPF1 function *in vivo* as is observed *in vitro*, we deem this unlikely given that mutation of the conserved residue in human UPF1 reduces RNA binding activity of the protein[2]. These data suggest that UPF2 likely plays a novel role in NMD and in regulating UPF1 function that is independent of its ability to induce conformational changes to UPF1 that stimulate its helicase activity.

Supplementary References

1. Franks, T.M., Signh, G. & Lykke-Andersen, J. UPF1 ATPase-dependent mRNP disassembly is required for completion of nonsense-mediated mRNA decay. *Cell* **143,** 938-50 (2010).

2. Lee, S.R., Pratt, G.A., Martinez, F.J., Yeo, G.W. & Lykke-Andersen, J. Target discrimination in nonsense-mediated mRNA decay requires UPF1 ATPase activity. *Mol. Cell* **59,** 413-25 (2015).

3. Weng, Y., Czaplinski, K. & Peltz, S.W. Genetic and biochemical characterization of mutations in the ATPase and helicase regions of the Upf1 protein. *Mol. Cell. Biol.* **16,** 5477-90 (1996).

4. He, F., Peltz, S.W., Donahue, J.L., Rosbash, M., & Jacobson, A. Stabilization and ribosome association of unspliced pre-mRNAs in a yeast *upf1*$^{-}$ mutant. *Proc. Natl. Acad. Sci. USA.* **90,** 7034-38 (1993).

5. Weng, Y., Czaplinski, K. & Peltz, S.W. ATP is a cofactor of the Upf1 protein that modulates its translation termination and RNA binding activities. *RNA.* **4,** 205-14 (1998).

6. Cheng, Z., Muhlrad, D., Lim, M.K., Parker, R., & Song, H. Structural and functional insights into the human Upf1 helicase core. *EMBO J.* **26,** 253-64 (2007).

7. Hu, W., Sweet, T.J., Chamnongpol, S., Baker, K.E. & Coller, J. Co-translational mRNA decay in Saccharomyces cerevisiae. *Nature* **461,** 225-9 (2009).

8. Pelechano, V., Wei, W. & Steinmetz, L.M. Widespread co-translational RNA decay reveals ribosome dynamics. *Cell* **161,** 1400-12 (2015).

9. Pisarev, A.V. *et al.* The role of ABCE1 in eukaryotic posttermination ribosome recycling. *Mol. Cell.* **37,** 196-210 (2010).

10. Shoemaker, C.J. & Green, R. Kinetic analysis reveals the ordered coupling of translation termination and ribosome recycling in yeast. *Proc. Natl. Acad. Sci. USA*. **108,** 1392-8 (2011).

11. Young, D.J., Guydosh, N.R., Zhang, F., Hinnebusch, A.G. & Green, R. Rli1/ABCE1 recycles terminating ribosomes and controls translation reinitiation in 3' UTRs in vivo. *Cell.* **162**, 872-84 (2015).

12. He, F., Brown, A.H. & Jacobson, A. Upf1p, Nmd2p, and Upf3p are interacting components of the yeast nonsense-mediated mRNA decay pathway. *Mol. Cell Biol.* **17**, 1580-94 (1997).

13. He, F., Ganesan, R. & Jacobson, A. Intra- and intermolecular regulatory interactions in Upf1, the RNA helicase central to nonsense-mediated mRNA decay in yeast. *Mol. Cell Biol.* **23,** 4672-84 (2013).

14. Chakrabarti, S. *et al.* Molecular mechanisms of the RNA-dependent ATPase activity of UPF1 and its regulation by Upf2. *Mol. Cell.* **41,** 693-703 (2011).

Supplementary Tables

Supplementary Table 1. Yeast strains used in this study

NAME	GENOTYPE	NOTES	REFERENCE
**yKB146**	MATa*, ura3, leu2, his3, met15, upf1::KanMX*	*upf1Δ*	EUROSCARF
**yKB154**	MATa*, ura3, leu2, his3, met15*	Wild-type	EUROSCARF
**yKB227 (yRP2076)**	MATa*, ura3, leu2, his3, met15, lys2, upf1::KanMX, xrn1::KanMX*	*upf1Δ/xrn1Δ*	Doma and Parker, 2006
**yKB478**	MATa*, ura3, leu2, his3, met15, upf1::MET15, upf3::KanMX*	*upf1Δ/upf3Δ*	This study
**yKB479**	MATa*, ura3, leu2, his3, met15, upf1::KanMX, upf2::MET15*	*upf1Δ/upf2Δ*	This study
**yKB494**	MATa*, ura3, leu2, his3, met15, UPF1:HA-HIS3*	HA tagged *UPF1*	This study
**yKB498**	MATa*, ura3, leu2, his3, met15, upf1::MET15 upf2::HIS3, upf3::KanMX*	*upf1Δ/upf2Δ/upf3Δ*	This study
**yKB704**	MATa*, ura3, leu2, his3, met15, HIS3-*P_{GAL1}*-3HA-RLI1*	HA tagged *RLI1* under control of *GAL1* promoter	This study

Supplementary Table 2. Plasmids used in this study

NAME	DESCRIPTION	NOTES	REFERENCE
**pFA6a-His3MX6-PGAL1-3HA**	Cassette used for tagging and placing *RLI1* under control of *GAL* promoter		Longtine et al., 1998
**pJC408**	*PGK1* with MS2 sites in 3' UTR, under control of *GAL1* promoter*; URA3, CEN*	Used for construction of pKB581, pKB589, pKB593.	Sweet et al., 2012
**pJC424**	*SL-PGK1* with MS2 sites, under control of *GAL1* promoter; *URA3, CEN*	Used for construction of pKB590	Sweet et al., 2012
**pKB102 (YEplac181)**	*LEU2, 2µ*	Used for construction of pKB598	Geitz and Sugino, 1988
**pKB105 (YCplac111)**	*LEU2, CEN*	Used for construction of pKB556	Geitz and Sugino, 1988
**pKB290**	*GFP* under control of *GAL1* promoter*; URA3, CEN*	Used for construction of pKB303, pKB311, pKB312	Baker and Parker, 2006
**pKB303**	*GFP-PTC 67* under control of *GAL1* promoter; *URA3, CEN*		Baker and Parker, 2006
**pKB311**	*GFP-PTC 135* under control of *GAL1* promoter; *URA3, CEN*		This study
**pKB312**	*GFP-PTC 200* under control of *GAL1* promoter; *URA3, CEN*		This study
**pKB509**	*GFP* under control of *TDH3* promoter; *URA3, CEN*	Used for construction of pKB510	This study
**pKB510**	*GFP-PTC 135* under control of *TDH3* promoter; *URA3, CEN*		This study
**pKB556**	*UPF1-HA; LEU2, CEN*		This study
**pKB576**	*UPF1-HA-DE572AA*; *LEU2, CEN*		This study
**pKB578**	*UPF1-HA-K436E*; *LEU2, CEN*		This study
**pKB579**	*UPF1-HA-RR793AA; LEU2, CEN*		This study
**pKB581**	*PGK1-PTC 142* with MS2 sites in 3' UTR, under control of *GAL1* promoter; *URA3, CEN*		This study
**pKB589**	*PGK1-PTC 344* with MS2 sites in 3' UTR, under control of *GAL1* promoter; *URA3, CEN*		This study
**pKB590**	*SL-PGK1-PTC142* with MS2 sites in 3' UTR, under control of *GAL1* promoter; *URA3, CEN*		This study
**pKB593**	*PGK1-PTC 225* with MS2 sites in 3' UTR, under control of *GAL1* promoter; *URA3, CEN*		This study
**pKB598**	*UPF1; LEU2, 2µ*		This study
**pKB607**	*UPF1-DE572AA; LEU2, 2µ*		This study
**pKB610**	*UPF1-DE572AA/RR793AA; LEU2, 2µ*		This study
**pKB621**	*UPF1-C62Y/DE572AA; LEU2, 2µ*		This study
**pKB638**	*UPF1-ΔCH; LEU2, 2µ*		This study
**pKB640**	*UPF1-F131E; LEU2, 2µ*		This study
**pKB641**	*UPF1-F131E/DE572AA; LEU2, 2µ*		This study
**pKB642**	*UPF1-C62Y; LEU2, 2µ*		This study

**pKB645**	*UPF1-ΔCH-DE572AA; LEU2, 2µ*		This study
**pKB690**	*UPF1-HA-K436P*; *LEU2, CEN*		This study
**pKB691**	*UPF1-HA-K436Q*; *LEU2, CEN*		This study
**pKB692**	*UPF1-HA-K436A*; *LEU2, CEN*		This study

Supplementary Table 3. Oligonucleotides used in this study

NAME	SEQUENCE	NOTES	REFERENCE
**oJC306**	GTCTAGCCGCGAGGAAGG	*SCR1* oligo probe	Smith et al., 2014
**oJC1006**	AGACATGGGTGATCCTCATG	*MS2* oligo probe; used as 3' UTR probe for *PGK1* reporters.	Sweet et al., 2012
**oKB147**	CCATACCTCTACCACCGGGGTGCTTTCTGTGCTTACCG	*CYH2* oligo probe	Meaux et al., 2008
**oKB245**	GGCCAACACTTGTCACTACTTTCACTTAAGGTGTTCAGTGCTTTTCAAGATACCCGGATC	Forward mutagenesis primer for inserting PTC at codon 67 of *GFP* mRNA	This study
**oKB246**	GGCCAACACTTGTCACTACTTTCACTTAAGGTGTTCAGTGCTTTTCAAGATACCCGGATC	Reverse mutagenesis primer for inserting PTC at codon 67 of *GFP* mRNA	This study
**oKB227**	GAGTTAAAAGGTATTGATTTTAAAGAAGATTAAAACATTCTTGGGCACAAATTGGAATAC	Forward mutagenesis primer for inserting PTC at codon 135 of *GFP* mRNA	This study
**oKB228**	GTATTCCAATTTGTGCCCAAGAATGTTTTAATCTTCTTTAAAATCAATACCTTTTAACTC	Reverse mutagenesis primer for inserting PTC at codon 135 of *GFP* mRNA	This study
**oKB229**	GGCCCTGTCCTTTTACCAGACAACCATTAACTGTCCACACAATCTGCCCTTTCGAAAG	Forward mutagenesis primer for inserting PTC at codon 200 of *GFP* mRNA	This study
**oKB230**	CTTTCGAAAGGGCAGATTGTGTGGACAGTTAATGGTTGTCTGGTAAAAGGACAGGGCC	Reverse mutagenesis primer for inserting PTC at codon 200 of *GFP* mRNA	This study
**oKB397**	CCACCAGGTGTTTTCGAATTCTAAAAGTTCGCTGCTGGTACTAAGG	Forward mutagenesis primer for inserting PTC at codon 344 of *PGK1* mRNA	This study
**oKB398**	CCTTAGTACCAGCAGCGAACTTTTAGAATTCGAAAACACCTGGTGG	Reverse mutagenesis primer for inserting PTC at codon 344 of *PGK1* mRNA	This study
**oKB454**	GAATTCGTGACGACGCGTAAGCTTGTCGACTTAGCAGCCAGATCCTTTGTATAG	*GFP* oligo probe	This study
**oKB823**	ACTAAATTTAGGACTGTGTTAATTGCTGCAAGTACTCAAGCTTCTGAGCCGG	Forward mutagenesis primer for UPF1-DE572AA mutation	This study
**oKB824**	CCGGCTCAGAAGCTTGAGTACTTGCAGCAATTAACACAGTCCTAAATTTAGT	Reverse mutagenesis primer for UPF1-DE572AA mutation	This study

**oKB825**	AATTCAAGGCCCACCAGGCACTGGTGA AACAGTTACTTCAGCAACGATTG	Forward mutagenesis primer for UPF1-K436E	This study
**oKB826**	CAATCGTTGCTGAAGTAACTGTTTCACC AGTGCCTGGTGGGCCTTGAATT	Reverse mutagenesis primer for UPF1-K436E mutation	This study
**oKB827**	AATGGTTTCTTACGTGATCCTGCTGCTC TAAACGTGGGTCTAACCCGTGC	Forward mutagenesis primer for UPF1-RR793AA mutation	This study
**oKB828**	GCACGGGTTAGACCCACGTTTAGAGCA GCAGGATCACGTAAGAAACCAAT	Reverse mutagenesis primer for UPF1-RR793AA mutation	This study
**oKB829**	CCTTCAGCTTCAGACAATTCATACGCGT ATTGTGGTATAGATTC	Forward mutagenesis primer for UPF1-C62Y mutation	This study
**oKB830**	GAATCTATACCACAATACGCGTATGAAT TGTCTGAAGCTGAAGG	Reverse mutagenesis primer for UPF1-C62Y mutation	This study
**oKB849**	CTACTGCAGTCACTAGCGAGTTCATTCT CCA	Forward PCR primer for amplification of *UPF1* ± 500 bp for making pKB598	This study
**oKB850**	CTAGGATCCGGCATAGTTCACACTTTTA TCTCC	Reverse PCR primer for amplification of *UPF1* ± 500 bp for making pKB598	This study
**oKB891**	GTCAAAAGGTCAAGGCTTCCTAGGAAG ATGTTCAAAAGTTC	Forward mutagenesis primer for inserting PTC at codon 142 of *PGK1* mRNA	This study
**oKB892**	GAACTTTTGAACATCTTCCTAGGAAGCC TTGACCTTTTGAC	Reverse mutagenesis primer for inserting PTC at codon 142 of *PGK1* mRNA	This study
**oKB908**	GATTCAATTGATTGACAACTAGTTGGAC AAGGTCGACTC	Forward mutagenesis primer for inserting PTC at codon 225 of *PGK1* mRNA	This study
**oKB909**	GAGTCGACCTTGTCCAACTAGTTGTCA ATCAATTGAATC	Reverse mutagenesis primer for inserting PTC at codon 225 of *PGK1* mRNA	This study
**oKB1031**	TCAGCTTCAGACAATTCAACTAGTTGTG CGTATTGTGGTATA	Forward mutagenesis primer for insertion of SpeI site at 5’ end of CH domain	This study
**oKB1032**	TATACCACAATACGCACAACTAGTTGAA TTGTCTGAAGCTGA	Reverse mutagenesis primer for insertion of SpeI site at 5’ end of CH domain	This study
**oKB1097**	GATCCAATAAAGACGCTACAACTAGTAT TAATGATATTGACGCCCCAGA	Forward mutagenesis primer for insertion of SpeI site at 3’ end of CH domain	This study
**oKB1098**	TCTGGGGCGTCAATATCATTAATACTAG TTGTAGCGTCTTTATTGGATC	Reverse mutagenesis primer for insertion of SpeI site at 3’ end of CH domain	This study
**oKB1123**	TTCAGCGCGAATTCCACCTAGCCTTCT GCAAAAGTTCTTAAGAAAACACGACAG ACCATAGAATTCGAGCTCGTTTAAAC	Forward PCR primer used for amplification of 3HA-GAL1 from pFA6a-His3MX6-PGAL1-3HA, with gene specific sequences for *RLI1*	This study
**oKB1124**	CTTTTTTGGTTTACATTTATCAGCGCTA ACGATAGCGATACGACTGTTTTTATCAC	Reverse PCR primer used for amplification of 3HA-	This study

	TCATGCACTGAGCAGCGTAATCTG	GAL1 from pFA6a-His3MX6-PGAL1-3HA, with gene specific sequences for *RLI1*	
**oKB1144**	CTGTGGACGTAAGAACGTGGAATTATT GGGATTTGTTTCCG	Forward mutagenesis primer for UPF1-F131E mutation	This study
**oKB1145**	CGGAAACAAATCCCAATAATTCCACGTT CTTACGTCCACAG	Reverse mutagenesis primer for UPF1-F131E mutation	This study
**oKB1156**	CTTAGCAACTGGAGCCAAAGA	*PGK1* oligo probe; used as 5' ORF probe for *PGK1* reporters.	This study
**oKB1324**	CCACCAGGCACTGGTGCAACAGTTACT TCAGC	Forward mutagenesis primer for UPF1-K436A mutation	This study
**oKB1325**	GCTGAAGTAACTGTTGCACCAGTGCCT GGTGG	Reverse mutagenesis primer for UPF1-K436A mutation	This study
**oKB1326**	CCACCAGGCACTGGTCCAACAGTTACT TCAGC	Forward mutagenesis primer for UPF1-K436P mutation	This study
**oKB1327**	GCTGAAGTAACTGTTGGACCAGTGCCT GGTGG	Reverse mutagenesis primer for UPF1-K436P mutation	This study
**oKB1328**	CCACCAGGCACTGGTCAAACAGTTACT TCAGC	Forward mutagenesis primer for UPF1-K436Q mutation	This study
**oKB1329**	GCTGAAGTAACTGTTTGACCAGTGCCT GGTGG	Reverse mutagenesis primer for UPF1-K436Q mutation	This study

Serdar *et al.* - Supplementary Figure 1

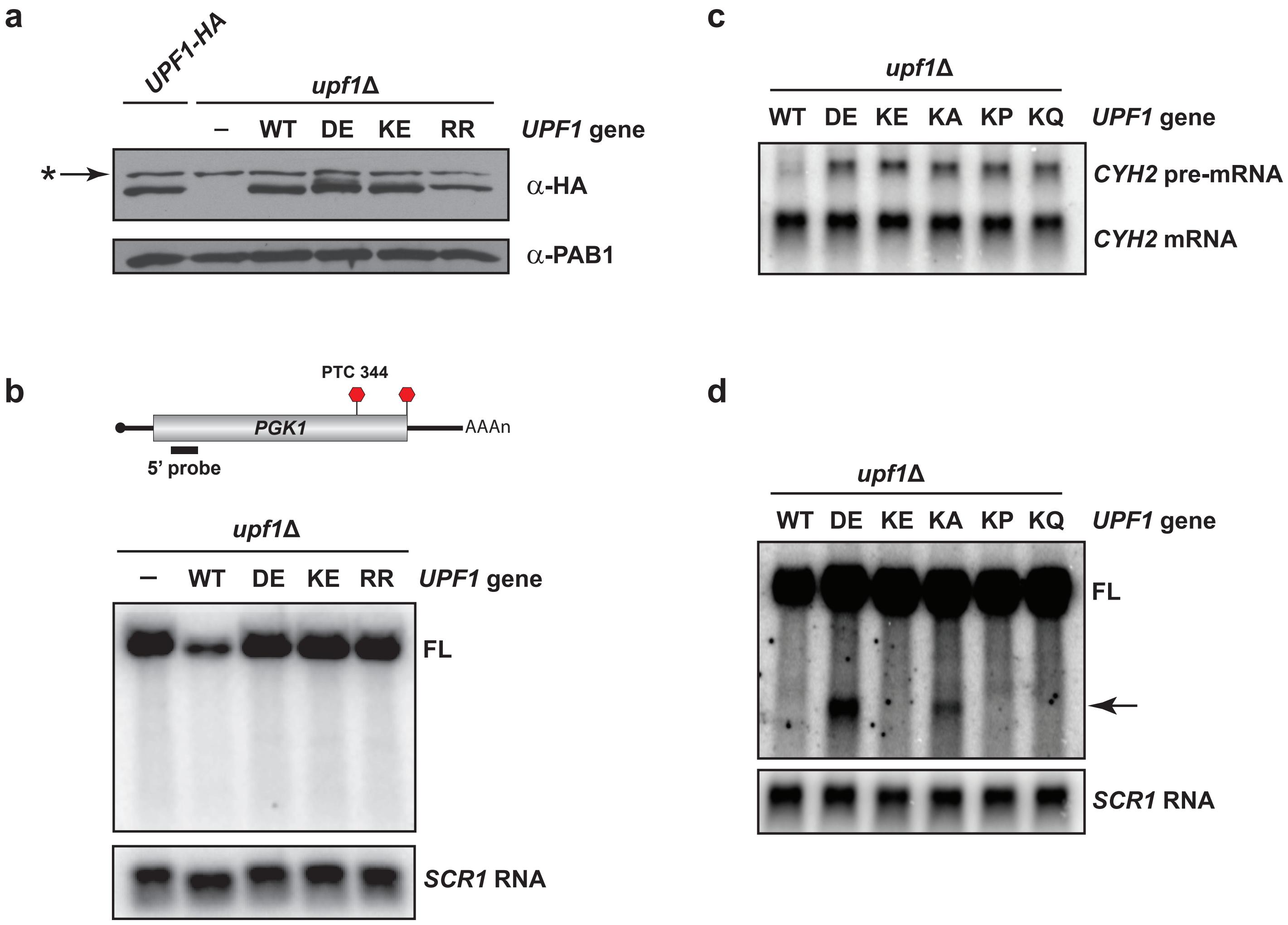

Supplementary Figure 1. 3' RNA decay fragments observed in ATPase-deficient UPF1 mutants are not generated in UPF1 ATP binding mutants with the exception of UPF1 K436A (**a**) Western blot analysis of epitope-tagged UPF1 expressed either from the chromosome (*UPF1-HA*) or from single-copy plasmids in *upf1Δ* cells. Protein levels were normalized to poly(A) binding protein (PAB1). A non-specific band cross-reacting with the α-HA antibody is indicated (asterisk). (**b**) Northern blot analysis of PTC-containing *PGK1* reporter mRNA in *upf1Δ* cells (-) complemented with wild-type or mutant *UPF1* detected using a probe complementary to a 5' region of the mRNA. (**c**) Northern analysis of *CYH2* RNA from *upf1Δ* cells expressing wild-type (WT), ATPase-deficient (DE), or putative ATP binding-deficient (KE, KA, KP, KQ) UPF1. NMD-sensitive *CYH2* pre-mRNA and NMD insensitive mRNA are indicated. (**d**) As in **c** but probing for *PGK1-PTC344* reporter mRNA. Full-length reporter mRNA (FL) and fragments (arrow) are indicated. RNA levels were normalized to NMD-insensitive *SCR1* mRNA.

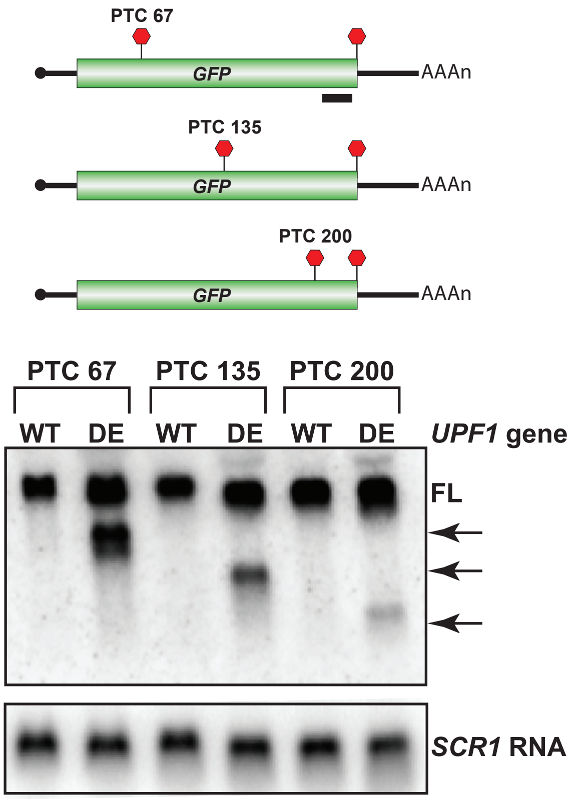

Supplementary Figure 2. 3' RNA fragments accumulate in a PTC position-dependent but substrate-independent manner

Northern analysis of *GFP* reporter mRNAs harboring PTCs at one of three indicated positions in *upf1*Δ cells expressing wild-type (WT) or ATPase-deficient (DE) UPF1. Full-length reporter mRNA (FL) and fragments (arrow) are indicated. RNA levels were normalized to NMD-insensitive *SCR1* mRNA.

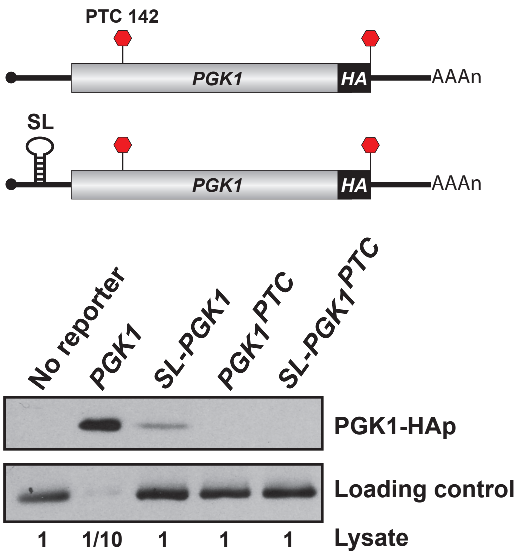

Supplementary Figure 3. Translation of *PGK1* reporter mRNA is strongly inhibited by insertion of a stable stemloop structure in the 5' UTR

Western blot analysis of epitope-tagged PGK1 expressed from normal or PTC-containing reporter genes either lacking (i.e. *PGK1* or *PGK1*^{PTC}) or harboring a stable stemloop structure (SL) in the 5' UTR (i.e. *SL-PGK1* or *SL-PGK1*^{PTC}). Based on sample loading (normalized to a loading control), translation of stemloop-containing *PGK1* mRNA was inhibited >100-fold; efficient termination at the PTC resulted in no detectable full-length protein.

Serdar *et al.* - Supplementary Figure 4

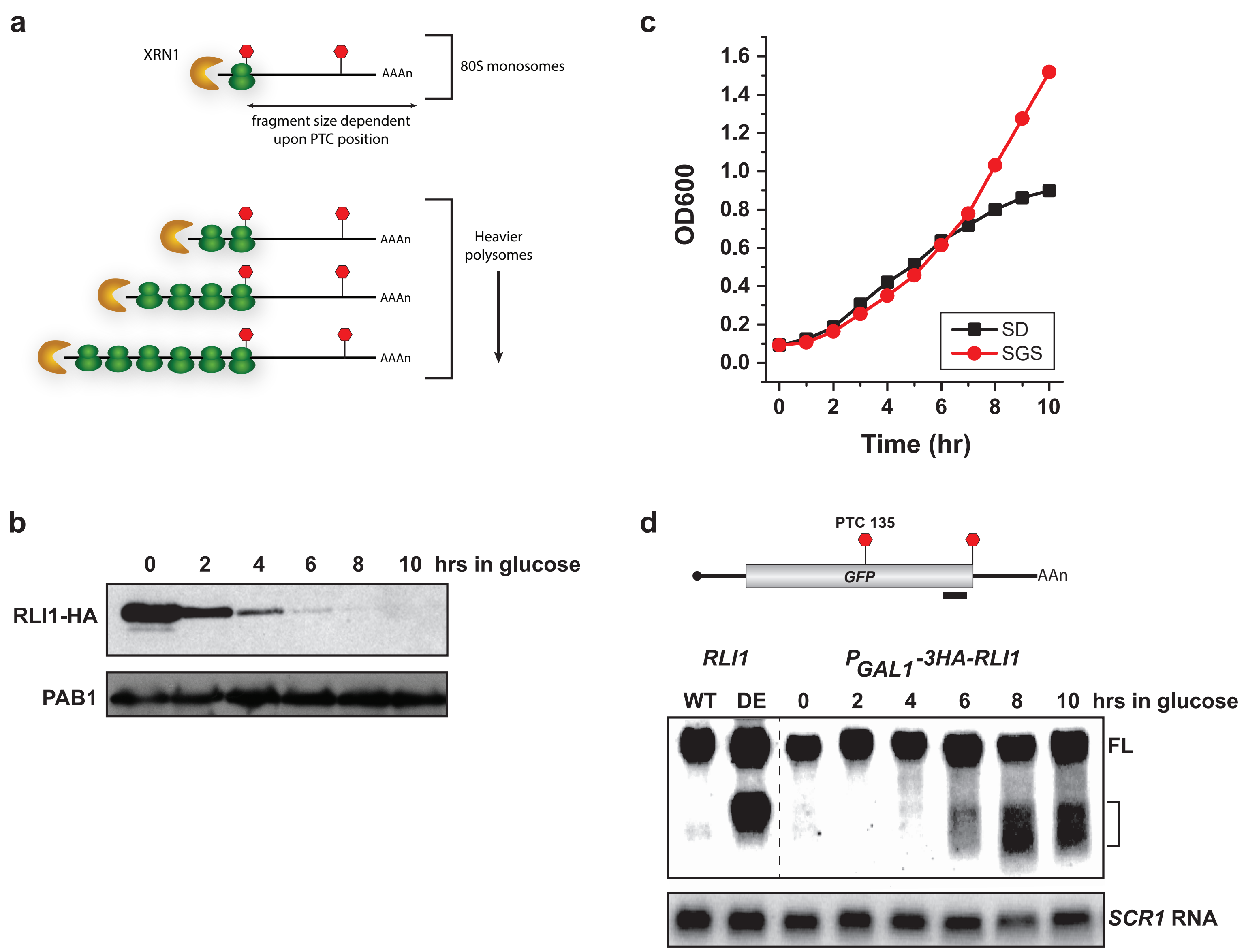

Supplementary Figure 4. Inhibition of translation termination induces formation of a 3' RNA decay fragment by co-translational mRNA decay

(**a**) Co-translational decay of PTC-containing mRNA inhibited for translation termination by ATPase-deficient UPF1 results in a build-up of ribosomes that present a greater 5' proximal block to XRN1 activity and the formation of increasingly larger 3' RNA decay fragments that co-sediment with heavier polyribosomes. (**b**) Western blot analysis of epitope-tagged RLI1 after transcriptional inhibition [in hours]. Protein levels were normalized to PAB1. (**c**) Growth of P_{GAL1}-*3HA-RLI1* cells under transcriptionally active (SGS, red line) or inactive (SD, black line) conditions. (**d**) Northern blot analysis of *GFP-PTC135* reporter mRNA in *upf1Δ* cells expressing wild-type (WT) or ATPase-deficient (DE) UPF1 (lanes 1 and 2), or P_{GAL1}-*3HA-RLI1* cells (expressing endogenous *UPF1*) after transcriptional inhibition for the indicated amount of time (lanes 3 – 8). RNA levels were normalized to NMD-insensitive *SCR1* RNA.

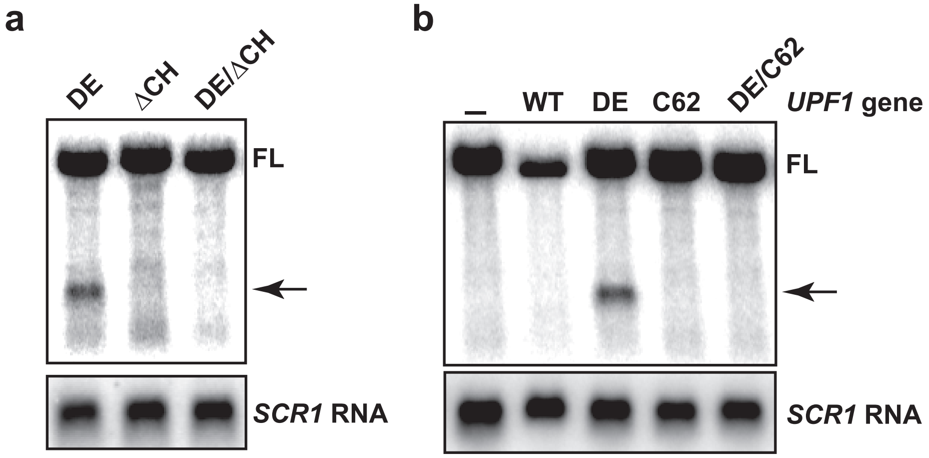

Supplementary Figure 5. Interaction with UPF2 is required for UPF1 function in premature translation termination

(**a**) Northern blot analysis of *PGK1-PTC344* reporter mRNA in *upf1Δ* cells expressing ATPase-deficient UPF1 (DE), UPF1 lacking the N-terminal cysteine/histidine rich domain (ΔCH), or UPF1 with both alterations (ΔCH/DE). (**b**) Northern blot analysis of *PGK1-PTC344* reporter mRNA in *upf1Δ* cells expressing ATPase-deficient UPF1 (DE), mutant UPF1 defective in binding to UPF2 (C62), or UPF1 with both mutations (C62/DE). Full-length (FL) reporter RNA and fragments (arrow) indicated. RNA levels were normalized to NMD-insensitive *SCR1* mRNA.

Uncropped Images Related to Figure 1

Figure 1b; PGK1

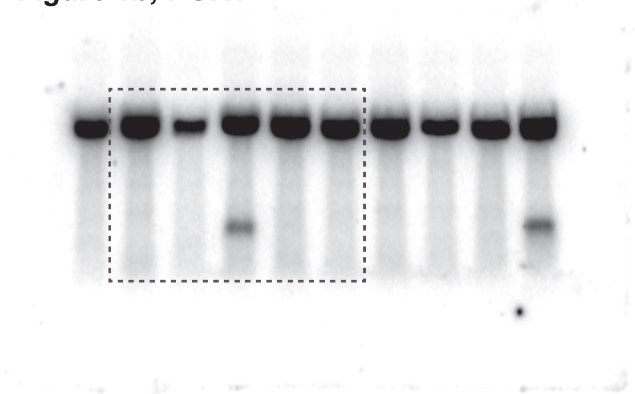

Figure 1b; SCR1

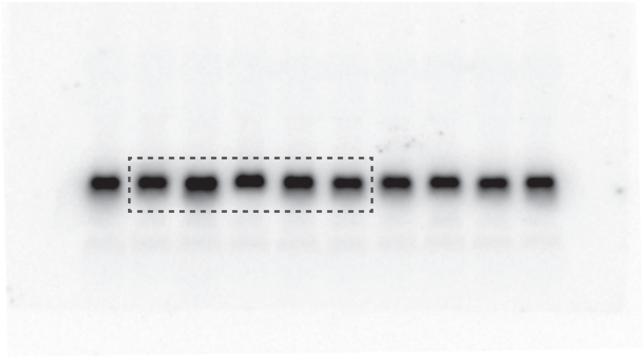

Figure 1d; PGK1

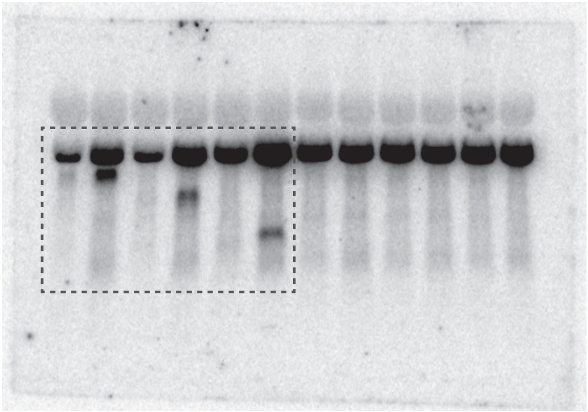

Figure 1d; SCR1

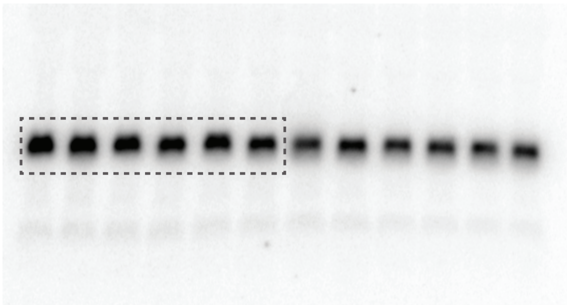

Figure 1c; PGK1

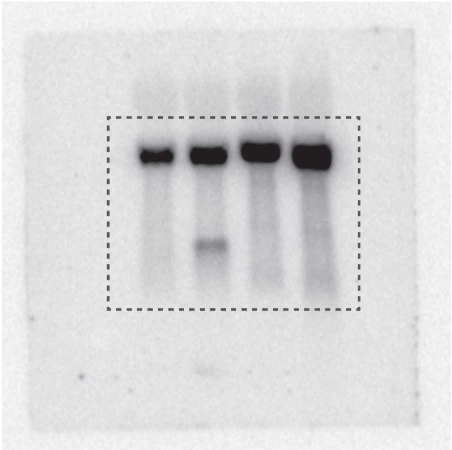

Figure 1c; SCR1

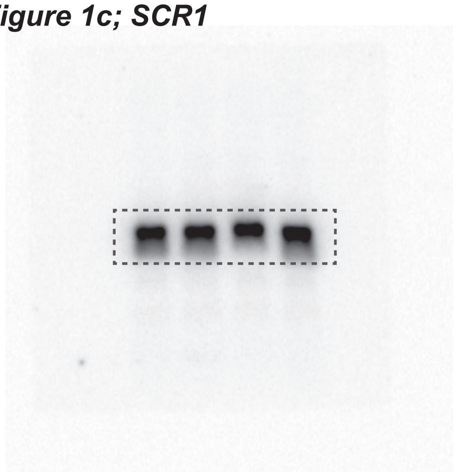

Uncropped Images Related to Figure 2

Figure 2a; PGK1

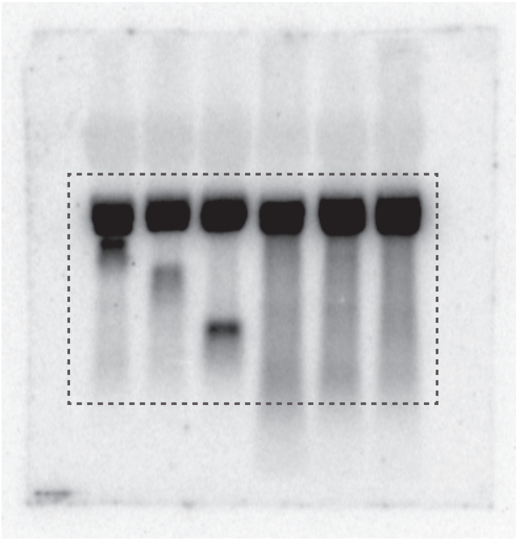

Figure 2b; PGK1

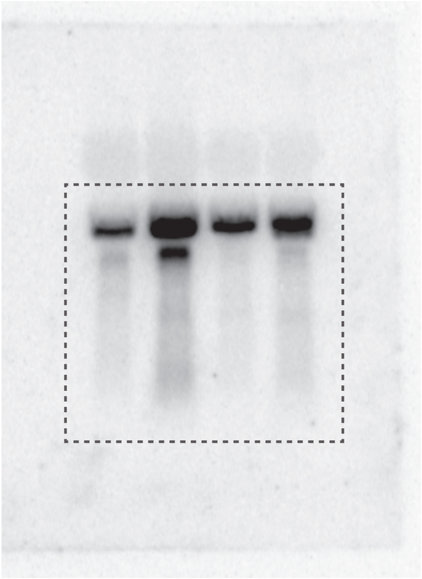

Figure 2a; SCR1

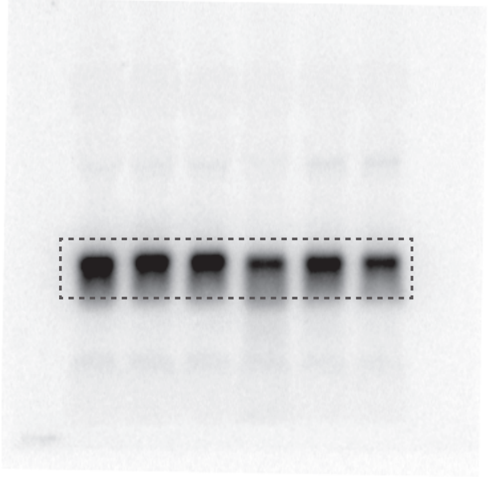

Figure 2b; SCR1

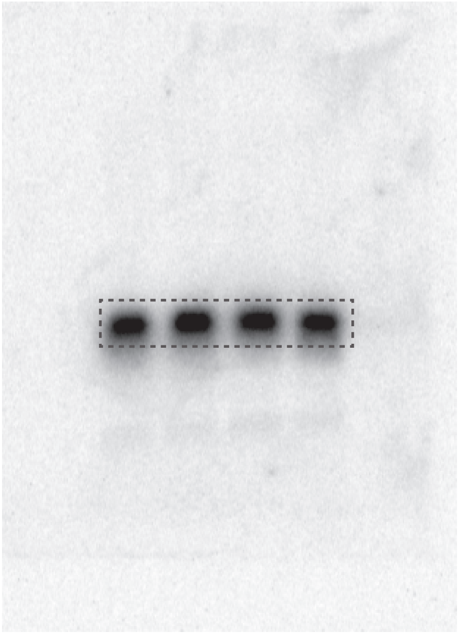

Uncropped Images Related to Figure 3

Figure 3b; PGK1

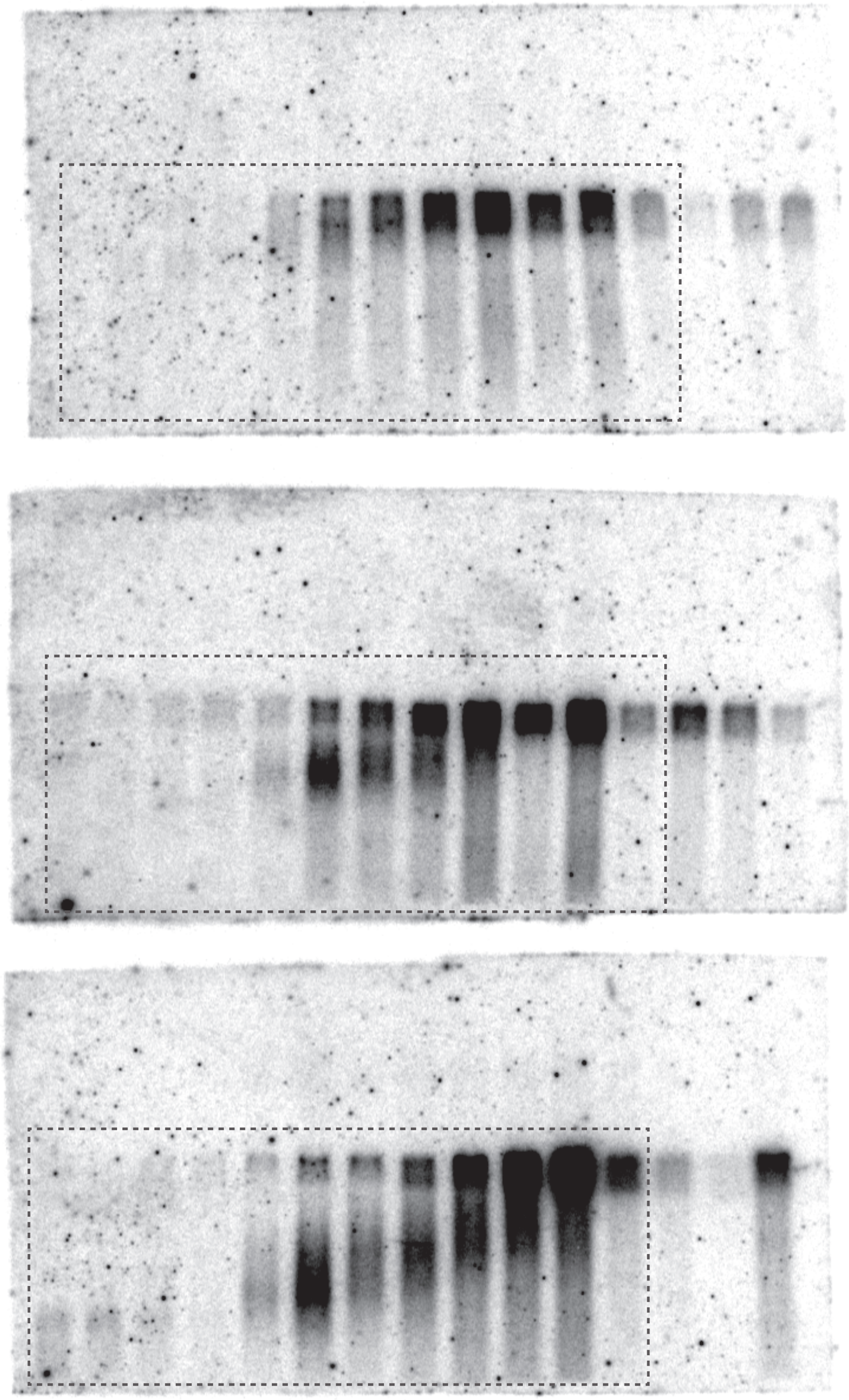

Uncropped Images Related to Figure 4

Figure 4a; PGK1

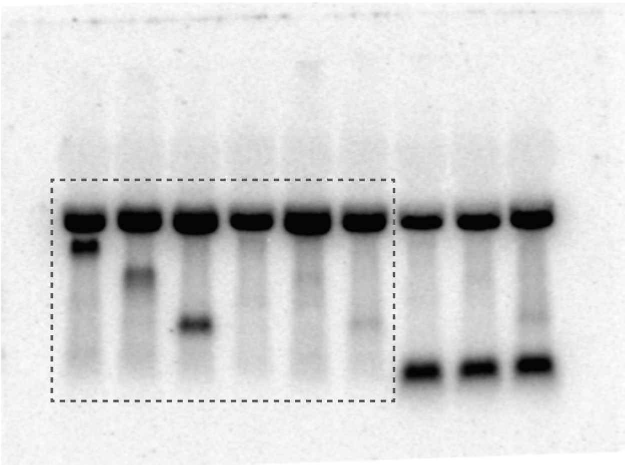

Figure 4a; SCR1

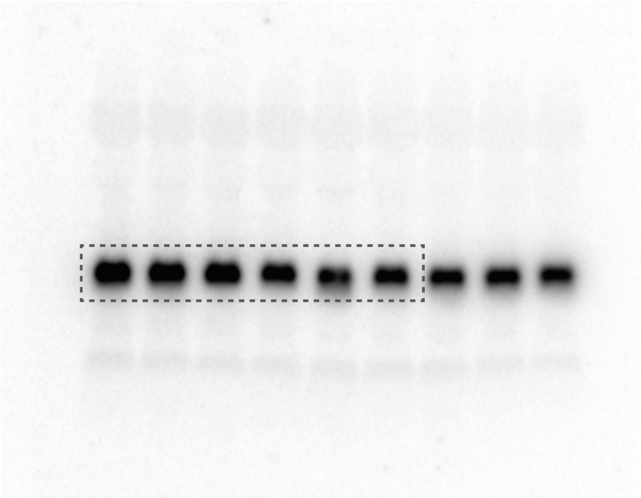

Figure 4b; PGK1

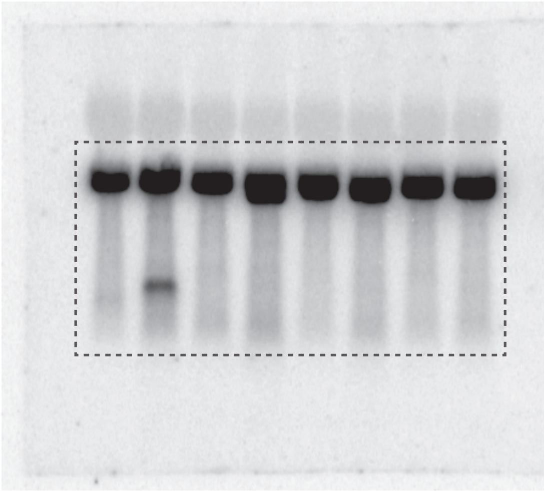

Figure 4b; SCR1

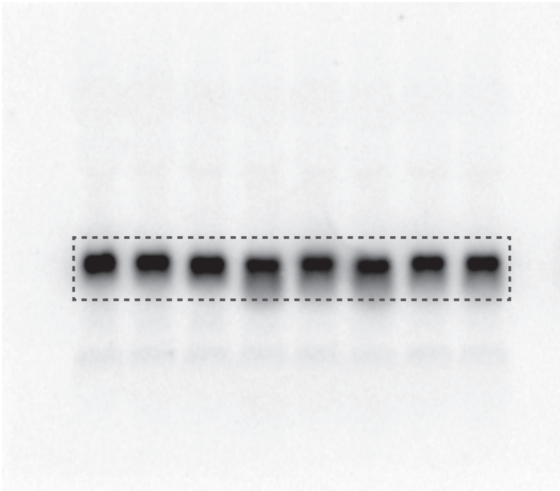

Figure 4c; PGK1

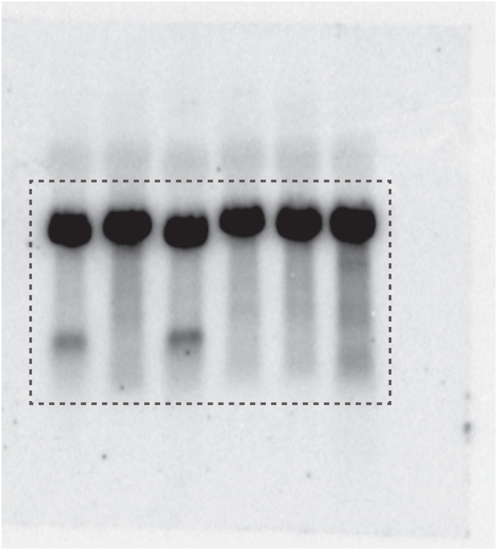

Figure 4c; SCR1

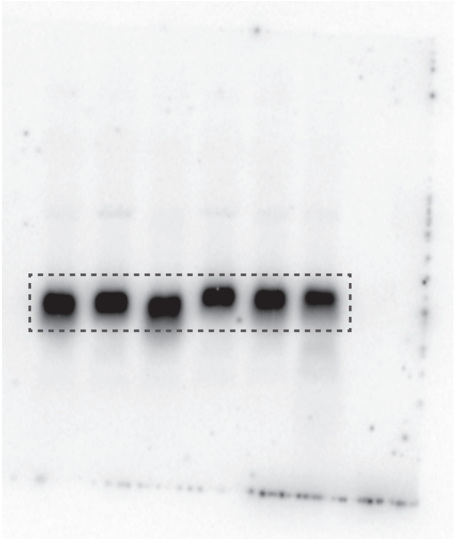

Supplement: Supplementary Information — Supplementary Figures, Supplementary Tables, Supplementary Discussion and Supplementary References [file ncomms14021-s1.pdf]
